# Primary peritoneal clear cell carcinoma treated with IMRT and interstitial HDR brachytherapy: a case report

**DOI:** 10.1120/jacmp.v15i1.4520

**Published:** 2014-01-06

**Authors:** Skyler B. Johnson, Joann I. Prisciandaro, Jessica Zhou, Scott W. Hadley, R. Kevin Reynolds, Shruti Jolly

**Affiliations:** ^1^ Department of Radiation Oncology University of Michigan Ann Arbor MI; ^2^ Department of Gynecologic Oncology University of Michigan Ann Arbor MI USA

**Keywords:** primary peritoneal clear cell carcinoma, brachytherapy, IMRT, radiation

## Abstract

Primary peritoneal clear cell carcinoma (PP‐CCC), which is a rare tumor with poor prognosis, is typically managed with surgery and/or chemotherapy. We present a unique treatment approach for a patient with a pelvic PP‐CCC, consisting of postchemotherapy intensity‐modulated radiation therapy (IMRT) followed by interstitial high‐dose–rate (HDR) brachytherapy. A 54‐year‐old female with an inoperable pelvic‐supravaginal 5.6 cm T3N0M0 PP‐CCC tumor underwent treatment with 6 cycles of carboplatin and taxol chemotherapy. Postchemotherapy PET/CT scan revealed a residual 3.3 cm tumor. The patient underwent CT and MR planning simulation, and was treated with 50 Gy to the primary tumor and 45 Gy to the pelvis including the pelvic lymph nodes, using IMRT to spare bowel. Subsequently, the patient was treated with an interstitial HDR brachytherapy implant, planned using both CT and MR scans. A total dose of 15 Gy in 5 Gy fractions over two days was delivered with Ir‐192 HDR brachytherapy. The total prescribed equivalent 2 Gy dose (EQD2) to the HDR planning target volume (PTV) from both the EBRT and HDR treatments ranged between 63 and $68.8\;{\text{Gy}}_{2}$ due to differential dosing of the primary and pelvic targets. The patient tolerated radiotherapy well, except for mild diarrhea not requiring medication. There was no patient‐reported acute toxicity one month following the radiotherapy course. At four months following adjuvant radiation therapy, the patient had near complete resolution of local tumor on PET/CT without any radiation‐associated toxicity. However, the patient was noted to have metastatic disease outside of the radiation field, specifically lesions in the liver and bone. This case report illustrates the feasibility of the treatment of a pelvic PP‐CCC with IMRT followed by interstitial HDR brachytherapy boost, which resulted in near complete local tumor response without significant morbidity.

PACS number: 87.55.‐x

## I. INTRODUCTION

Primary peritoneal clear cell carcinoma (PP‐CCC) is extremely rare, accounting for approximately 3% of primary peritoneal carcinomas (PPC) with an incidence of 0.46 per 100,000.^1^, ^2^, ^3^ Other PPCs include serous adenocarcinoma, peritoneal serous borderline tumor, serous papillary adenocarcinoma, and mesotheliomas.^(4)^ These tumors are histologically similar to ovarian tumors and are believed to behave similarly.^4^, ^5^ Therefore, treatment for PPC has historically reflected this belief, utilizing debulking surgery followed by chemotherapy or chemotherapy and second‐look surgery.^(3)^ However, mortality remains high, with a median survival of approximately 24 months^6^, ^7^, ^8^, ^9^ and five‐year survival rate of 18%.^(10)^ Unfortunately, most studies on outcomes of PPC do not include PP‐CCC.^6^, ^7^, ^8^, ^11^ To date, there are only nine reported cases of PP‐CCC in the English medical literature, none of which were treated with radiation.^12^, ^13^, ^14^, ^15^, ^16^, ^17^, ^18^, ^19^ In those cases reporting outcomes, prognosis was much poorer than has been seen in retrospective studies of PPC, with 33% (two of six cases) mortality within six months, and 100% mortality within six months in those with residual disease following initial therapy.^(3)^ New treatment strategies may be necessary to improve local control and decrease mortality for patients with PP‐CCC.

This case report describes treatment of an inoperable PP‐CCC with adjuvant radiation that did not achieve complete resolution following chemotherapy. The tumor was treated with intensity‐modulated radiation therapy (IMRT) to the pelvis followed by intraoperative interstitial catheter placement and high‐dose‐rate (HDR) brachytherapy. Both CT and MR simulations were performed for clear delineation of the tumor and organs at risk (OARs) for planning purposes prior to both external‐beam radiation therapy (EBRT) and HDR treatment. Specifically, the gross tumor volume (GTV) and the OARs (e.g., rectum, bladder, and bowel) were delineated.

## II. CASE REPORT

**Figure 1:**
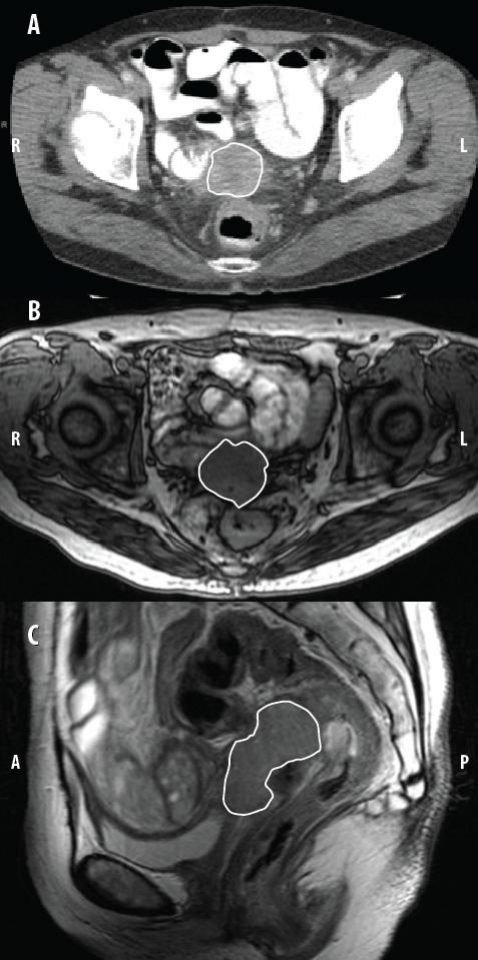
Diagnostic imaging: (a) axial CT image of the pelvis obtained at presentation shows a supravaginal 3.7×3.9cm primary peritoneal clear cell carcinoma (PP‐CCC) and an (b) axial and (c) sagittal T2‐weighted MR image of the pelvis obtained one month following presentation which shows a supravaginal 5.6×3.7×3.5cm primary peritoneal clear cell carcinoma. The lesion appears inseparable from the anterior wall of the proximal rectum.

A 54‐year‐old Japanese Gl P0 female was referred to the University of Michigan Comprehensive Cancer Center (UMCCC) for examination and review of a 3.7×3.9cm high‐grade PP‐CCC by CT scan and vaginal biopsy confirmation by her gynecologist. Her gynecologic history was significant for three myomectomies, and a total abdominal hysterectomy and bilateral salpingo‐oopherectomy for uterine leiomyomas and menorrhagia, 15 years prior to presentation. At initial presentation, the patient complained of a three‐month history of pelvic discomfort, which she described as pressure and constipation, along with early satiety, fatigue, and a ten‐pound weight loss. A vaginal biopsy was performed, which showed high‐grade clear cell adenocarcinoma. PAP smear at the time was reported as atypical glandular cells of undetermined significance (AGUS). The patient then underwent completion staging workup, including CT scan of the abdomen and pelvis, which showed a 3.7cm×3.9cm soft tissue mass in the deep pelvis (Fig. 1(a)).

On initial consultation with Radiation Oncology prior to the initiation of chemotherapy, the patient reported slight vaginal bleeding since biopsy, as well as persistent fatigue. Pelvic exam revealed an irregular, firm, polypoid, friable lesion involving the entire horizontal extent of the vaginal apex, which extended inferiorly to the upper one‐third of the vagina. Rectal exam showed an approximately 4 cm length of abutment of the anterior aspect of the rectum. The rectovaginal septum was intact and there was no palpable lymphadenopathy. One month following initial presentation and CT, an MRI of the pelvis confirmed the presence of a 5.6×3.7×3.5cm mass on the proximal vagina and vaginal cuff, which appeared to be inseparable from the anterior wall of the proximal rectum and rectosigmoid junction, likely representing local invasion (Figs. 1(b) and 1(c)).

**Figure 2:**
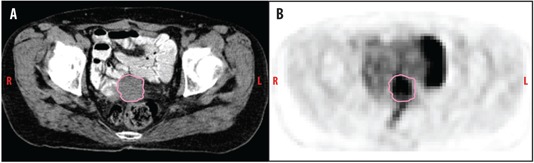
Postchemotherapy (a) axial CT and (b) PET image of the pelvis shows a residual 3.3 cm prerectal/primary peritoneal clear cell carcinoma with FDG activity in the posterior vagina.

One month following initial presentation, the patient completed six cycles of carboplatin and taxol chemotherapy over the next four months. Following chemotherapy, a ^18^F‐FDG PET/CT scan revealed a 3.3 cm prerectal soft tissue mass with FDG activity in the posterior vagina consistent with active neoplasm (Fig. 2).

The patient then underwent MR simulation and on the following day, a CT simulation, in the Department of Radiation Oncology. For both the MR and CT simulations, the patient was positioned supine on a foam pad with legs straight and feet banded together. To improve the visualization of the vaginal apex and vault, a 2 cm diameter radiopaque vaginal marker was inserted at time of each simulation (ShadowForm, Izi Medical Products, Owings Mills, MD). The MR simulation was then performed using a Siemens Skyra 3T scanner (Siemens Healthcare Diagnostics, Inc., Erlangen, Germany). The following MR scans were acquired: T2‐weighted TSE axial, coronal, and sagittal images at 3 mm slice thickness, T1‐weighted TSE coronal images with large field of view at 4 mm slice thickness, T1‐weighted TSE axial images at 3 mm slice thickness, and postgadolinium T1‐weighted axial, sagittal, and coronal images at 3 mm slice thickness. The T1W images were acquired to assist with nodal volume delineation, and the T2W images were used to define the gross and microscopic disease. The CT scan was performed using a 16 slice Philips Brilliance CT scanner (Royal Philips Electronics, Eindhoven, Netherlands). Images were acquired from the top of the T10 vertebral body to 5 cm inferior of the ischial tuberosities with 3 mm slice thickness.

**Figure 3:**
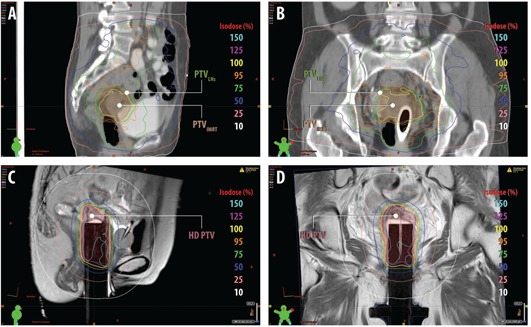
Isodose distributions on the (a) parasagittal and (b) paracoronal CT view for the approved external beam radiotherapy, and (c) parasagittal and (d) paracoronal T2‐weighted MR view for the approved HDR brachytherapy treatment plan. The external beam ${\text{PTV}}_{\text{IMRT}}$ and ${\text{PTV}}_{\text{LNs}}$ volumes are shown in the shadowed red and green contours, and the HDR PTV volume is shown in the shadowed red contour.

**Figure 4:**
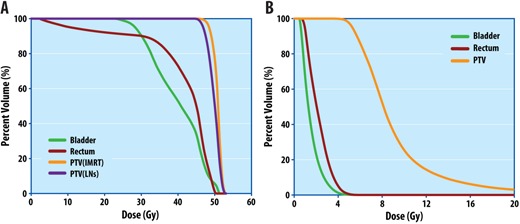
Cumulative dose‐volume histograms for (a) external beam and (b) high‐dose‐rate brachytherapy plans.

A treatment plan was generated for external beam radiotherapy based on the CT images. The CT and MR images were not registered for the EBRT plan. The structures were contoured by the physician on the CT images, using anatomic guidance from the MR, as well as the PET/CT for the delineation of gross disease. One month following completion of chemotherapy, the patient began a course of EBRT with a nine‐field coplanar 16 MV IMRT plan with gantry angles spanning from $20^{\circ }–340^{\circ }$ (IEC coordinate system) at 40° intervals. The treatment plan was designed to deliver a total dose of 50 Gy in 2 Gy daily fractions to the primary tumor $\left({\text{PTV}}_{\text{IMRT}}=\text{CTV}+1\;\text{cm uniform margin}\right)$ and 45 Gy in 1.8 Gy fractions to the pelvis including the pelvic lymph nodes $\left({\text{PTV}}_{\text{LNs}}={\text{CTV}}_{\text{LNs}}+1\;\text{cm uniform margin}\right)$. The dose distribution for the approved plan is shown in parasagittal and paracoronal view in Figs. 3(a) and 3(b), respectively. The objectives of the IMRT plan was to deliver at least 95% of the prescription dose to the PTV with a uniformity of ±5% while minimizing dose to the organs at risk (OARs). The dose constraints to the OARs were: bowel max dose ≤50Gy (in 2 Gy fractions), V45≤25%; femoral head V30≤20%; rectum V50≤50%; bladder ALARA. IMRT treatment planning was performed with an in‐house treatment planning system, UMPlan. The cumulative dose‐volume histograms (DVHs) for ${\text{PTV}}_{\text{IMRT}},{\text{PTV}}_{\text{LNs}}$, bladder, and rectum are presented in Fig. 4(a).

**Figure 5:**
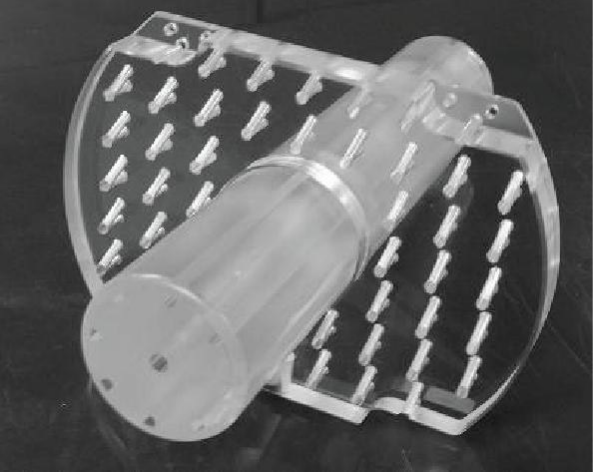
A custom 30 millimeter diameter interstitial vaginal cylinder with perineal template.

One week following EBRT, the patient received an HDR brachytherapy boost. For the interstitial HDR brachytherapy boost, the patient was taken to the operating room and underwent a minilaparotomy and placement of the interstitial applicator by the gynecologic oncologist, as recommended per the American Brachytherapy Society guidelines for interstitial brachytherapy.^(20)^ The patient was examined, prepped, and draped in the low anterior lithotomy position with a Foley catheter inserted and radiocontrast injected into the balloon. A custom 30 mm HDR interstitial cylindrical applicator was then placed into the vagina and a custom perineal template was sutured into place (Fig. 5). Although not used, the perineal template allows for the insertion of interstitial needles either perpendicular to the template or at a 15° angle from normal incidence, which may be desirable in the case of pubic arch interference. A minilaparotomy and omental J‐flap were performed. The omental J‐flap allowed for increased distance between the interstitial needles and surrounding bowel. Nine interstitial needles were manually inserted into the cylinder, including eight along the periphery and one in the center of the vaginal cylinder. Following the implant procedure, the patient underwent CT and MR simulation in the department of Radiation Oncology with the same imaging units detailed above. Prior to both simulation scans, the Foley catheter was tugged to ensure the balloon was positioned at the bladder neck. The CT scan was performed from the L4/L5 interspace to 5 cm inferior of the ischial tuberosities with 1 mm slice thickness. To minimize applicator displacement, the patient was transferred to a detachable MR couch using a slide board. The following MR scans were acquired based on GEC‐ESTRO recommendations:^(21)^ T2 TSE axial, coronal, and sagittal images at 3 mm slice thickness, 3D T2 (SPC) sagittal images at 0.9 mm slice thickness, 3D T1 (MPRAGE) sagittal images at 0.9 mm slice thickness, and postgadolinium T1 TSE axial, coronal, and sagittal images at 3 mm slice thickness.

**Figure 6:**
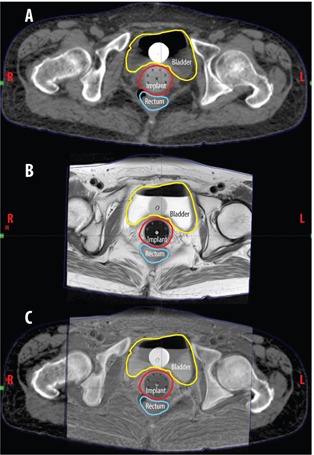
CT (a), T2‐weighted MR (b), and registered CT/MR (c) axial images of the custom interstitial brachytherapy cylinder at time of planning simulation. The yellow, red, and blue outlines represent the bladder, PTV, and rectum contours, respectively.

**Figure 7:**
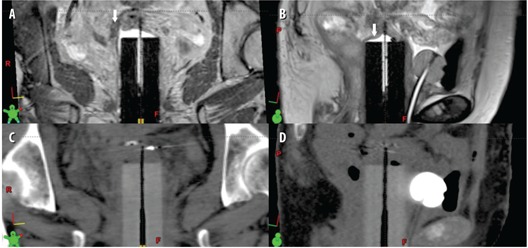
Paracoronal ((a) and (c)) and parasagittal ((b) and (d)) T2‐weighted MR and CT, respectively, through the custom interstitial applicator.

Following the MR simulation, the patient was transferred to an HDR suite on the MR detachable couch as the HDR treatment plan was developed. The CT and MR images were imported into a commercial brachytherapy planning system, BrachyVision 8.9 (Varian Medical Systems, Palo Alto, CA). The CT and MR images were then manually aligned based on the position of the cylindrical vaginal applicator in BrachyVision. The alignment was visually evaluated by comparing the position of the cylindrical applicator and the neighboring anatomy between the CT and MR images. The clinical target volume (${\text{CTV}}_{\text{HDR}}$) was drawn on the CT scan using the superimposed T2‐weighted axial MR images, and the interstitial needles were digitized employing the CT dataset, based on evidence of gross residual disease and areas of close proximity intraoperatively. The ${\text{PTV}}_{\text{HDR}}$ volume was equivalent to the ${\text{CTV}}_{\text{HDR}}$ volume. Figure 6 shows representative CT, MR (T2‐weighted 3D), and registered CT/MR images (using the T2‐weighted 3D image) at the level of midcylinder. Figure 7 illustrates the difference between soft tissue on the CT and MR scans, as well as the visualization of the applicator channels, in both paracoronal and parasagittal images. An HDR treatment plan was designed to deliver 5 Gy per fraction to a minimum of 95% of the ${\text{PTV}}_{\text{HDR}}(V100\;({\text{PTV}}_{\text{HDR}})\geq 95\%)$, while minimizing dose to the rectum and bladder. The final dose was determined by the cumulative tolerable doses to the normal critical structures, including small bowel, bladder, and rectum. The dose distribution for the approved plan is shown on parasagittal and paracoronal MR T2W images in Figs. 3(c) and 3(d), respectively. Additionally, the cumulative dose‐volume histograms (DVHs) for PTV(HDR), bladder, and rectum are presented in Fig. 4(b). Following planning, Ir‐192 HDR brachytherapy was used to deliver 15 Gy in 5 Gy fractions over two days. Each fraction was at least six hours apart to allow for normal tissue repair, and was delivered with a GammaMedPlus iX afterloader (Varian Medical Systems, Palo Alto, CA).

**Figure 8:**
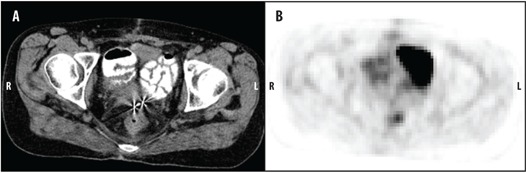
Postradiotherapy CT (a) and PET image (b) of the pelvis obtained four months following radiotherapy shows near complete resolution of tumor.

At one‐month follow‐up there were no signs of acute toxicity. The patient did report mild vaginal discomfort and pelvic pain, however denied fatigue, nausea, abdominal pain, incontinence, vaginal discharge, and blood per rectum or vagina. Her pretreatment constipation had resolved and she was passing one to two stools per day. At four months follow‐up, the patient underwent PET/CT imaging which revealed near complete resolution of tumor within the radiation fields (Fig. 8). Unfortunately, the imaging also revealed multiple new lesions not seen on the initial PET/CT, including metastases to the bone and liver.

## III. DISCUSSION

This is the first reported case regarding the benefit of radiation in the treatment of an inoperable PP‐CCC. Following six cycles of carboplatin and taxol chemotherapy and incomplete tumor resolution, IMRT was delivered to the primary tumor and pelvic lymph nodes followed by an interstitial HDR brachytherapy boost. Although the posttreatment PET/CT imaging revealed metastatic disease, the radiation therapy resulted in local tumor control, defined as no evidence of disease recurrence within the treatment field, and limited treatment morbidity.

PP‐CCC is a rare disease with poor patient outcomes. Currently, there are no retrospective studies or consensus agreements on the appropriate therapy. Case reports provide limited data, as there are only nine reports in the English medical literature.^12^, ^13^, ^14^, ^15^, ^16^, ^17^, ^18^, ^19^ Although debulking surgery,^12^, ^15^, ^17^, ^18^ as well as debulking surgery with chemotherapy,^14^, ^16^, ^17^, ^19^ were used in these reports, outcomes remained suboptimal with two of six patients dying within six months^17^, ^19^ and one recurrence at 32 months.^(14)^ The other cases remained disease free at 6 months, 12 months, and 20 months following completion of treatment.^16^, ^17^, ^18^ Both cases of death occurred within six months when the patients had residual tumors of >2cm, whereas the other cases had no evidence of residual tumor following treatment.^(3)^ In our case, the patient presented with inoperable disease that was inseparable from the anterior wall of the proximal rectum and rectosigmoid on MR. She also had residual disease on PET/CT scan following chemotherapy. Inoperable or residual gross disease requires higher doses of radiation to the pelvis, which can also result in increased GI or GU toxicity including pain, bleeding, bladder irritation, and diarrhea. Interstitial HDR brachytherapy is an ideal treatment option because it optimizes radiation dose to the gross tumor while limiting dose to the adjacent normal tissue. Furthermore, the intraoperative approach of HDR catheter placement with minilaparotomy allows for ideal catheter placement within the residual tumor and displacement of bowel, which may increase local control and decrease morbidity.^22^, ^23^ Intraoperative catheter placement has less surgical morbidity compared with debulking surgery. Nearly one‐quarter of patients may experience major complications following surgery, such as invasive radiologic intervention, reoperation, unplanned ICU admission, chronic disability, or death,^(24)^ and more than one‐third of women older than 75 have morbidity following debulking surgery.^(25)^ Additionally, CT and MR simulations were performed on the patient prior to both EBRT and HDR, and were used to help delineate the GTV/CTV (for EBRT), the CTV (for HDR), and the organs at risk. We achieved a V95 of 96.5% and 98.7% to the ${\text{PTV}}_{\text{IMRT}}\;(\text{CTV}+1\;\text{cm uniform margin})$ and ${\text{PTV}}_{\text{HDR}}$, respectively, and the total prescribed equivalent 2 Gy dose (EQD2[EQD2=Bioeffective dose/(1+(2/(α/β))]) to ${\text{PTV}}_{\text{HDR}}$ ranged from 63 to $68.8\;{\text{Gy}}_{2}$ due to differential dosing of the primary and pelvic targets, assuming an α/β of ten. The D2cc (most exposed 2 cm^3^) of the bladder and the rectum was 50.5 Gy and 49.3 Gy, respectively, for EBRT and 11.8 Gy and 11.9 Gy, respectively, for the HDR treatment plan. This resulted in an EQD2 of $50.7\;{\text{Gy}}_{2}$ and $16.5\;{\text{Gy}}_{2}$ for the bladder and $49.0\;{\text{Gy}}_{2}$ and $16.7\;{\text{Gy}}_{2}$ for the rectum with the EBRT and HDR, respectively, assuming an α/β of three (Table 1). Perioperative interstitial catheter placement and CT and MR‐based planning allowed for dose optimization and resulted in decreased morbidity and improved local control.

Although there are no retrospective studies of PP‐CCC, there is evidence to suggest that EBRT may improve local control for women with clear cell histology associated with uterine and ovarian carcinoma. Adjuvant EBRT has been shown to improve overall survival in patients with uterine clear cell carcinoma (UCCC) in a recent retrospective review.^(26)^ Thomas et al.^(27)^ conducted a multi‐institutional review of 99 patients with UCCC and concluded that adjuvant EBRT improved progression‐free survival (67% vs. 36%), and reduced pelvic sidewall (18% vs. 53%) and vaginal recurrences (7% vs. 35%) for those at risk of local failure. There is also evidence that HDR brachytherapy improves local control and improves outcomes in endometrial^28^, ^29^ and uterine^30^, ^31^ clear cell cancer types. Radiation remains an effective treatment that produces tumor resolution, as identified in case reports of clear cell histology with recurrent^(32)^ and chemotherapy‐resistant^(33)^ ovarian clear cell carcinoma. In this report, postradiation therapy PET/CT revealed near complete tumor response, showing that this treatment approach was effective. Concurrent systemic therapy may have limited metastatic progression, although there is evidence that PP‐CCC tumors are resistance to conventional platinum‐based chemotherapies,^(17)^ suggesting the need for novel therapies.

We attempted to control local micrometastatic progression through treatment of the pelvic lymph nodes using an initial course of IMRT. The patient did have distant metastatic progression four months following adjuvant radiation. However, she did experience resolution of gross tumor, with no evidence of local progression. For this patient, brachytherapy was the ideal treatment solution because of the location of the tumor, which was near the proximal vagina, and because residual tumor disease requires high doses of radiation. Interstitial HDR brachytherapy catheter placement and CT/MR planning allowed for dose optimization to the primary tumor. The patient tolerated the procedure well, reporting no postoperative morbidity and minimal acute radiation related side effects.

**Table 1:** Summary of the dose quality parameters for the EBRT (${\text{PTV}}_{\text{IMRT}}$) and HDR treatment plans

			${\text{EQD}}_{2}\;(\text{Gy}2)$ ^a^	
*Dose Quality Parameter*	*EBRT*	*HDR*	*EBRT*	*HDR*
PTV V100 (%)	61.60	97.36	—	—
PTV V95 (%)	96.54	98.68	—	—
PTV V90 (%)	99.98	99.42	—	—
PTV D100 (Gy)	44.22	9.45	43.4	10.4
PTV D95 (Gy)	47.74	15.81	47.4	20.1
PTV D90 (Gy)	48.40	17.1	48.1	22.4
Bladder D2cc (Gy)	50.49	11.85	50.7	16.5
Bladder point^b^ (Gy)	N/A	13.02	—‐	19.1
Rectum D2cc (Gy)	49.28	11.94	49.0	16.7

a ^a^ The equivalent 2 Gy dose, ${\text{EQD}}_{2}$, has been calculated assuming an α/β of ten for the PTV and three for the bladder and rectum.

b ^b^ The bladder point was positioned based on the recommendations of ICRU 38.(34)

## V. CONCLUSIONS

While surgery and chemotherapy remain the mainstay for treatment of PP‐CCC, radiotherapy for local control appears to be effective in local control of PP‐CCC. Advancements in imaging and radiation techniques may make it possible to deliver radiation to residual areas of disease without causing excessive morbidity.

## ACKNOWLEDGMENTS

The authors would like to thank Dr. Yue Cao, Dr. James Balter, and Jeremy French for their guidance and assistance with developing the appropriate MRI protocols used for both the EBRT and brachytherapy components of this study.

## Supporting information

Supplementary Material
