# Supplementary material for: Primary peritoneal clear cell carcinoma treated with IMRT and interstitial HDR brachytherapy: a case report
Source: J Appl Clin Med Phys. 2014 Jan 6;15(1):202–12. doi: 10.1120/jacmp.v15i1.4520 (PMC5711230; doi:10.1120/jacmp.v15i1.4520)
Supplement: Supplementary file 1 — Supplementary Material [file ACM2-15-202-s001.docx]

Primary peritoneal clear cell carcinoma treated with IMRT and interstitial HDR brachytherapy: a case report

**Skyler B. Johnson BS^1^, Joann I. Prisciandaro PhD^1^, Jessica Zhou MD^1^, Scott W. Hadley PhD^1^, R. Kevin Reynolds MD^2^, Shruti MD^1^**

*Department of Radiation Oncology,***^1^** *University of Michigan*

*Department of Gynecologic Oncology,***^2^** *University of Michigan*

Corresponding author:

Shruti Jolly MD

*University of Michigan Medical Center
1500 East Medical Center Drive
Ann Arbor, MI 48109
Tel.: (734) 936 7810
Fax: (734) 763 7370*
Email: shrutij@med.umich.edu

Submitted: May 2, 2013

Accepted: August 1, 2013

Running title: Peritoneal Clear Cell Treated with IMRT and HDR Brachytherapy

The author has no financial disclosures or conflicts of interest.

**Abstract:**

**Background:**

Primary peritoneal clear cell carcinoma (PP-CCC), which is a rare tumor with poor prognosis, is typically managed with surgery and/or chemotherapy. We present a unique treatment approach for a patient with a pelvic PP-CCC, consisting of post-chemotherapy intensity modulated radiation therapy (IMRT) followed by interstitial high-dose rate (HDR) brachytherapy.

**Methods and Materials:**

A 54-year-old female with an inoperable pelvic-supravaginal 5.6 cm T3N0M0 PP-CCC tumor underwent treatment with 6 cycles of carboplatin and taxol chemotherapy. Post-chemotherapy PET/CT scan revealed a residual 3.3 cm tumor. The patient underwent CT and MR planning simulation, and was treated with 50 Gy to the primary tumor and 45 Gy to the pelvis including the pelvic lymph nodes, using IMRT to spare bowel. Subsequently, the patient was treated with an interstitial HDR brachytherapy implant, planned using both CT and MR scans. A total dose of 15 Gy in 5 Gy fractions over 2 days was delivered with Ir-192 HDR brachytherapy. The total prescribed equivalent 2 Gy dose (EQD2) to the HDR planning target volume (PTV) from both the EBRT and HDR treatments ranged between 63 and 68.8 Gy_2_ due to differential dosing of the primary and pelvic targets.

**Results:**

The patient tolerated radiotherapy well except for mild diarrhea not requiring medication. There was no patient reported acute toxicity one month following the radiotherapy course. At four months following adjuvant radiation therapy, the patient had near complete resolution of local tumor on PET/CT without any radiation-associated toxicity. However, the patient was noted to have metastatic disease outside of the radiation field, specifically lesions in the liver and bone.

**Conclusions:**

This case report illustrates the feasibility of the treatment of a pelvic PP-CCC with IMRT followed by interstitial HDR brachytherapy boost, which resulted in near complete local tumor response without significant morbidity.

*Keywords: Primary peritoneal clear cell carcinoma; Brachytherapy; IMRT; Radiation*

*PACS code: Radiation therapy, treatment strategy in, 87.55.-x*

**Introduction**

Primary peritoneal clear cell carcinoma (PP-CCC) is extremely rare, accounting for approximately 3% of primary peritoneal carcinomas (PPC) with an incidence of 0.46 per 100,000 ([1-3](#_ENREF_1)). Other PPCs include serous adenocarcinoma, peritoneal serous borderline tumor, serous papillary adenocarcinoma and mesotheliomas ([4](#_ENREF_4)). These tumors are histologically similar to ovarian tumors and are believed to behave similarly ([4](#_ENREF_4), [5](#_ENREF_5)). Therefore, treatment for PPC has historically reflected this belief, utilizing debulking surgery followed by chemotherapy or chemotherapy and second look surgery ([3](#_ENREF_3)). However, mortality remains high with a median survival of approximately 24 months ([6-9](#_ENREF_6)) and 5-year survival rate of 18% ([10](#_ENREF_10)). Unfortunately, most studies on outcomes of PPC do not include PP-CCC ([6-8](#_ENREF_6), [11](#_ENREF_11)). To date, there are only 9 reported cases of PP-CCC in the English medical literature, none of which were treated with radiation ([12-19](#_ENREF_12)). In those cases reporting outcomes, prognosis was much poorer than has been seen in retrospective studies of PPC, with 33% (2 of 6 cases) mortality within 6 months and 100% mortality within 6 months in those with residual disease following initial therapy ([3](#_ENREF_3)). New treatment strategies may be necessary to improve local control and decrease mortality for patients with PP-CCC.

This case report describes treatment of an inoperable PP-CCC with adjuvant radiation that did not achieve complete resolution following chemotherapy. The tumor was treated with intensity modulated radiation therapy (IMRT) to the pelvis followed by intra-operative interstitial catheter placement and high-dose rate (HDR) brachytherapy. Both CT and MR simulations were performed for clear delineation of the tumor and organs at risk (OARs) for planning purposes prior to both external beam radiation therapy (EBRT) and HDR treatment. Specifically, the gross tumor volume (GTV) and the OARs (e.g. rectum, bladder and bowel) were delineated.

**Case report**

A 54-year-old Japanese G1 P0 female was referred to the University of Michigan Comprehensive Cancer Center (UMCCC), for examination and review of a 3.7 x 3.9 cm high-grade PP-CCC by CT scan and vaginal biopsy confirmation by her gynecologist. Her gynecologic history was significant for 3 myomectomies and a total abdominal hysterectomy and bilateral salpingo-oopherectomy for uterine leiomyomas and menorrhagia, 15-years prior to presentation. At initial presentation, the patient complained of a 3-month history of pelvic discomfort, which she described as pressure and constipation, along with early satiety, fatigue and a 10-pound weight loss. A vaginal biopsy was performed, which showed high-grade clear cell adenocarcinoma. PAP smear at the time was reported as atypical glandular cells of undetermined significance (AGUS). The patient then underwent completion staging workup including CT scan of the abdomen and pelvis, which showed a 3.7-cm x 3.9-cm soft tissue mass in the deep pelvis (Fig. 1 A).

On initial consultation with Radiation Oncology, prior to the initiation of chemotherapy, the patient reported slight vaginal bleeding since biopsy as well as persistent fatigue. Pelvic exam revealed an irregular, firm, polypoid, friable lesion involving the entire horizontal extent of the vaginal apex, which extended inferiorly to the upper one third of the vagina. Rectal exam showed an approximately 4 cm length of abutment of the anterior aspect of the rectum. The rectovaginal septum was intact and there was no palpable lymphadenopathy. One month following initial presentation and CT, an MRI of the pelvis confirmed the presence of a 5.6 x 3.7 x 3.5 cm mass on the proximal vagina and vaginal cuff, which appeared to be inseparable from the anterior wall of the proximal rectum and rectosigmoid junction, likely representing local invasion (Fig. 1 B & C).

One month following initial presentation, the patient completed 6-cycles of carboplatin and taxol chemotherapy over the next four months. Following chemotherapy, a 18F-FDG PET/CT scan revealed a 3.3 cm prerectal soft tissue mass with FDG activity in the posterior vagina consistent with active neoplasm (Fig. 2A & 2B).

The patient then underwent MR simulation and on the following day, a CT simulation, in the Department of Radiation Oncology. For both the MR and CT simulations, the patient was positioned supine on a foam pad with legs straight and feet banded together. To improve the visualization of the vaginal apex and vault, a 2 cm diameter radiopaque vaginal marker was inserted at time of each simulation (ShadowForm, Izi Medical Products, Owings Mills, MD). The MR simulation was then performed using a Siemens Skyra 3T scanner (Siemens Healthcare Diagnostics, Inc., Erlangen, Germany). The following MR scans were acquired: T2-weighted TSE axial, coronal, and sagittal images at 3 mm slice thickness, T1-weighted TSE coronal images with large field of view at 4 mm slice thickness, T1-weighted TSE axial images at 3 mm slice thickness, and post-gadolinium T1-weighted axial, sagittal, and coronal images at 3 mm slice thickness. The T1W images were acquired to assist with nodal volume delineation, and the T2W images were used to define the gross and microscopic disease. The CT scan was performed using a 16 slice, Philips Brilliance CT scanner (Royal Philips Electronics, Eindhoven, Netherlands). Images were acquired from the top of the T10 vertebral body to 5 cm inferior of the ischial tuberosities with 3 mm slice thickness.

A treatment plan was generated for external beam radiotherapy based on the CT images. The CT and MR images were not registered for the EBRT plan. The structures were contoured by the physician on the CT images, using anatomic guidance from the MR as well as the PET/CT for the delineation of gross disease. One month following completion of chemotherapy, the patient began a course of EBRT with a nine field coplanar 16MV IMRT plan with gantry angles spanning from 20 – 340^o^ (IEC coordinate system) at 40^o^ intervals. The treatment plan was designed to deliver a total dose of 50 Gy in 2 Gy daily fractions to the primary tumor (PTV_IMRT_ = CTV + 1 cm uniform margin) and 45 Gy in 1.8 Gy fractions to the pelvis including the pelvic lymph nodes (PTV_LNs_ = CTV_LNs_ + 1 cm uniform margin). The dose distribution for the approved plan is shown in parasagittal and paracoronal view in Figure 3a and 3b, respectively. The objectives of the IMRT plan was to deliver at least 95% of the prescription dose to the PTV with a uniformity of +/- 5% while minimizing dose to the organs at risk (OARs). The dose constraints to the OARs were: bowel max dose ≤ 50Gy (in 2 Gy fractions), V45 ≤ 25%; femoral head V30 ≤ 20%; rectum V50 ≤ 50%; bladder ALARA. IMRT treatment planning was performed with an in-house treatment planning system, UMPlan. The cumulative dose volume histograms (DVHs) for PTV_IMRT_, PTV_LNs_, bladder and rectum are presented in Figure 4a.

One week following EBRT, the patient received an HDR brachytherapy boost. For the interstitial HDR brachytherapy boost, the patient was taken to the operating room and underwent a mini-laparotomy and placement of the interstitial applicator with the gynecologic oncologist as recommended per the American Brachytherapy Society guidelines for interstitial brachytherapy ([20](#_ENREF_20)). The patient was examined, prepped, and draped in the low anterior lithotomy position with a Foley catheter inserted and radiocontrast injected into the balloon. A custom 30 mm HDR interstitial cylindrical applicator was then placed into the vagina and a custom perineal template was sutured into place (Figure 5). Although not used, the perineal template allows for the insertion of interstitial needles either perpendicular to the template or at a 15^o^ angle from normal incidence which may be desirable in the case of pubic arch interference. A mini-laparotomy and omental J-flap were performed. The omental J-flap allowed for increased distance between the interstitial needles and surrounding bowel. Nine interstitial needles were manually inserted into the cylinder, including 8 along the periphery and 1 in the center of the vaginal cylinder. Following the implant procedure, the patient underwent CT and MR simulation in the department of Radiation Oncology with the same imaging units detailed above. Prior to both simulation scans, the Foley catheter was tugged to ensure the balloon was positioned at the bladder neck. The CT scan was performed from the L4/L5 interspace to 5 cm inferior of the ischial tuberosities with 1 mm slice thickness. To minimize applicator displacement, the patient was transferred to a detachable MR couch using a slide board. The following MR scans were acquired based on GEC-ESTRO recommendations ([21](#_ENREF_21)): T2 TSE axial, coronal, and sagittal images at 3 mm slice thickness, 3D T2 (SPC) sagittal images at 0.9 mm slice thickness, 3D T1 (MPRAGE) sagittal images at 0.9 mm slice thickness, and post-gadolinium T1 TSE axial, coronal, and sagittal images at 3 mm slice thickness.

Following the MR simulation, the patient was transferred to an HDR suite on the MR detachable couch as the HDR treatment plan was developed. The CT and MR images were imported into a commercial brachytherapy planning system, BrachyVision^TM^ 8.9 (Varian Medical Systems, Palo Alto, CA,). The CT and MR images were then manually aligned based on the position of the cylindrical vaginal applicator in BrachyVision^TM^. The alignment was visually evaluated by comparing the position of the cylindrical applicator and the neighboring anatomy between the CT and MR images. The clinical target volume (CTV_HDR_) was drawn on the CT scan using the superimposed T2 weighted axial MR images, and the interstitial needles were digitized employing the CT dataset, based on evidence of gross residual disease and areas of close proximity intraoperatively. The PTV_HDR_ volume was equivalent to the CTV_HDR_ volume. Figure 6 shows representative CT, MR (T2 weighted 3D), and registered CT/MR images (using the T2 weighted 3D image) at the level of mid cylinder. Figure 7 illustrates the difference between soft tissue on the CT and MR scans, as well as the visualization of the applicator channels, in both paracoronal and parasagittal images. An HDR treatment plan was designed to deliver 5 Gy per fraction to a minimum of 95% of the PTV_HDR_ (V100 (PTV_HDR_) ≥ 95%) while minimizing dose to the rectum and bladder. The final dose was determined by the cumulative tolerable doses to the normal critical structures, including small bowel, bladder and rectum. The dose distribution for the approved plan is shown on parasagittal and paracoronal MR T2W images in Figures 3c and 3d, respectively. Additionally, the cumulative dose volume histograms (DVHs) for PTV(HDR), bladder and rectum are presented in Figure 4b. Following planning, Ir-192 HDR brachytherapy was used to deliver 15 Gy in 5 Gy fractions over 2 days. Each fraction was at least 6 hours apart to allow for normal tissue repair and was delivered with a GammaMedPlus iX afterloader (Varian Medical Systems, Palo Alto, CA).

At 1 month follow-up there were no signs of acute toxicity. The patient did report mild vaginal discomfort and pelvic pain, however denied fatigue, nausea, abdominal pain, incontinence, vaginal discharge and blood per rectum or vagina. Her pretreatment constipation had resolved and she was passing 1- 2 stools per day. At 4 months follow up, the patient underwent PET/CT imaging which revealed near complete resolution of tumor within the radiation fields (Fig. 8). Unfortunately, the imaging also revealed multiple new lesions not seen on the initial PET/CT, including metastases to the bone and liver.

**Discussion**

This is the first reported case regarding the benefit of radiation in the treatment of an inoperable PP-CCC. Following 6 cycles of carboplatin and taxol chemotherapy and incomplete tumor resolution, IMRT was delivered to the primary tumor and pelvic lymph nodes followed by an interstitial HDR brachytherapy boost. Although the post-treatment PET/CT imaging revealed metastatic disease, the radiation therapy resulted in local tumor control, defined as no evidence of disease recurrence within the treatment field, and limited treatment morbidity.

PP-CCC is a rare disease with poor patient outcomes. Currently, there are no retrospective studies or consensus agreements on the appropriate therapy. Case reports provide limited data as there are only 9 reports in the English medical literature ([12-19](#_ENREF_12)). Although debulking surgery ([12](#_ENREF_12), [15](#_ENREF_15), [17](#_ENREF_17), [18](#_ENREF_18)) as well as debulking surgery with chemotherapy ([14](#_ENREF_14), [16](#_ENREF_16), [17](#_ENREF_17), [19](#_ENREF_19)) were used in these reports, outcomes remained suboptimal with 2 of 6 patients dying within 6 months ([17](#_ENREF_17), [19](#_ENREF_19)) and 1 recurrence at 32 months ([14](#_ENREF_14)). The other cases remained disease free at last follow up of 6, 12 and 20 months ([16-18](#_ENREF_16)). Both cases of death occurred within 6 months when the patients had residual tumors of > 2 cm, whereas the other cases had no evidence of residual tumor following treatment ([3](#_ENREF_3)). In our case, the patient presented with inoperable disease that was inseparable from the anterior wall of the proximal rectum and rectosigmoid on MR. She also had residual disease on PET/CT scan following chemotherapy. Inoperable or residual gross disease requires higher doses of radiation to the pelvis, which can also result in increased GI or GU toxicity including pain, bleeding, bladder irritation and diarrhea. Interstitial HDR brachytherapy is an ideal treatment option because it optimizes radiation dose to the gross tumor, while limiting dose to the adjacent normal tissue. Furthermore, the intraoperative approach of HDR catheter placement with mini-laparotomy allows for ideal catheter placement within the residual tumor and displacement of bowel, which may increase local control and decrease morbidity ([22](#_ENREF_22), [23](#_ENREF_23)). Intraoperative catheter placement has less surgical morbidity compared with debulking surgery. Nearly one-quarter of patients may experience major complications following surgery such as invasive radiologic intervention, re-operation, unplanned ICU admission, chronic disability, or death ([24](#_ENREF_24)) and more than one-third of women greater than 75 have morbidity following debulking surgery ([25](#_ENREF_25)). Additionally, CT and MR simulations were performed on the patient prior to both EBRT and HDR, and were used to help delineate the GTV/CTV (for EBRT), the CTV (for HDR), and the organs at risk. We achieved a V95 of 96.5 and 98.7% to the PTV_IMRT_ (CTV + 1 cm uniform margin) and PTV_HDR_, respectively, and the total prescribed equivalent 2 Gy dose (EQD2 [EQD2=Bioeffective dose/(1+(2/(α/β))]) to PTV_HDR_ ranged from 63 to 68.8 Gy_2_ due to differential dosing of the primary and pelvic targets, assuming an α/β of 10. The D2cc (most exposed 2 cm^3^) of the bladder and the rectum was 50.5 Gy and 49.3 Gy, respectively, for EBRT and 11.8 Gy and 11.9 Gy, respectively, for the HDR treatment plan. This resulted in an EQD2 of 50.7 Gy_2_ and 16.5 Gy_2_ for the bladder and 49.0 Gy_2_ and 16.7 Gy_2_ for the rectum with the EBRT and HDR, respectively, assuming an α/β of 3 (Table 1). Perioperative interstitial catheter placement and CT and MR based planning allowed for dose optimization and resulted in decreased morbidity and improved local control.

Although there are no retrospective studies of PP-CCC, there is evidence to suggest that EBRT may improve local control for women with clear cell histology associated with uterine and ovarian carcinoma. Adjuvant EBRT has been shown to improve overall survival in patients with uterine clear cell carcinoma (UCCC) in a recent retrospective review ([26](#_ENREF_26)). Thomas *et al.* ([27](#_ENREF_27)), conducted a multi-institutional review of 99 patients with UCCC and concluded that adjuvant EBRT improved progression-free survival (67% vs. 36%), and reduced pelvic sidewall (18% vs. 53%) and vaginal recurrences (7% vs. 35%) for those at risk of local failure. There is also evidence that HDR brachytherapy improves local control and improves outcomes in endometrial ([28](#_ENREF_28), [29](#_ENREF_29)) and uterine ([30](#_ENREF_30), [31](#_ENREF_31)) clear cell cancer types. Radiation remains an effective treatment that produces tumor resolution as identified in case reports of clear cell histology with recurrent ([32](#_ENREF_32)) and chemotherapy-resistant ([33](#_ENREF_33)) ovarian clear cell carcinoma. In this report, post-radiation therapy PET/CT revealed near complete tumor response, showing that this treatment approach was effective. Concurrent systemic therapy may have limited metastatic progression, although, there is evidence that PP-CCC tumors are resistance to conventional platinum based chemotherapies ([17](#_ENREF_17)) suggesting the need for novel therapies.

We attempted to control local micrometastatic progression through treatment of the pelvic lymph nodes using an initial course of IMRT. The patient did have distant metastatic progression four months following adjuvant radiation. However, she did experience resolution of gross tumor with no evidence of local progression. For this patient, brachytherapy was the ideal treatment solution because of the location of the tumor, which was near the proximal vagina and because residual tumor disease requires high doses of radiation. Interstitial HDR brachytherapy catheter placement and CT/MR planning allowed for dose optimization to the primary tumor. The patient tolerated the procedure well, reporting no postoperative morbidity and minimal acute radiation related side effects.

**Conclusion**

While surgery and chemotherapy remains the mainstay for treatment of PP-CCC, radiotherapy for local control appears to be effective in local control of PP-CCC. Advancements in imaging and radiation techniques may make it possible to deliver radiation to residual areas of disease without causing excessive morbidity.

**Acknowledgements**

The authors would like to thank Dr. Yue Cao, Dr. James Balter, and Jeremy French for their guidance and assistance with developing the appropriate MRI protocols used for both the EBRT and brachytherapy components of this study.

**References**

1. Moll UM, Valea F, Chumas J. Role of p53 alteration in primary peritoneal carcinoma. Int J Gynecol Pathol1997 Apr;16(2):156-62.

2. Goodman MT, Shvetsov YB. Rapidly increasing incidence of papillary serous carcinoma of the peritoneum in the United States: fact or artifact? Int J Cancer2009 May 1;124(9):2231-5.

3. Wuntakal R, Lawrence A. Are oestrogens and genetic predisposition etiologic factors in the development of clear cell carcinoma of the peritoneum? Med Hypotheses2013 Feb;80(2):167-71.

4. Fox H. Primary neoplasia of the female peritoneum. Histopathology1993 Aug;23(2):103-10.

5. Jaaback KS, Ludeman L, Clayton NL, Hirschowitz L. Primary peritoneal carcinoma in a UK cancer center: comparison with advanced ovarian carcinoma over a 5-year period. Int J Gynecol Cancer2006 Jan-Feb;16 Suppl 1:123-8.

6. Barda G, Menczer J, Chetrit A, Lubin F, Beck D, Piura B, Glezerman M, Modan B, Sadetzki S. Comparison between primary peritoneal and epithelial ovarian carcinoma: a population-based study. Am J Obstet Gynecol2004 Apr;190(4):1039-45.

7. Fromm GL, Gershenson DM, Silva EG. Papillary serous carcinoma of the peritoneum. Obstet Gynecol1990 Jan;75(1):89-95.

8. Killackey MA, Davis AR. Papillary serous carcinoma of the peritoneal surface: matched-case comparison with papillary serous ovarian carcinoma. Gynecol Oncol1993 Nov;51(2):171-4.

9. Roh SY, Hong SH, Ko YH, Kim TH, Lee MA, Shim BY, Byun JH, Woo IS, Kang JH, Hong YS, Lee KS. Clinical characteristics of primary peritoneal carcinoma. Cancer Res Treat2007 Jun;39(2):65-8.

10. Nam JH, Kim YM, Jung MH, Kim KR, Yoo HJ, Kim DY, Kim JH, Kim YT, Mok JE. Primary peritoneal carcinoma: experience with cytoreductive surgery and combination chemotherapy. Int J Gynecol Cancer2006 Jan-Feb;16(1):23-8.

11. Bloss JD, Brady MF, Liao SY, Rocereto T, Partridge EE, Clarke-Pearson DL. Extraovarian peritoneal serous papillary carcinoma: a phase II trial of cisplatin and cyclophosphamide with comparison to a cohort with papillary serous ovarian carcinoma-a Gynecologic Oncology Group Study. Gynecol Oncol2003 Apr;89(1):148-54.

12. Evans H, Yates WA, Palmer WE, Cartwright RL, Antemann RW. Clear cell carcinoma of the sigmoid mesocolon: a tumor of the secondary mullerian system. Am J Obstet Gynecol1990 Jan;162(1):161-3.

13. Hama Y, Iwasaki Y, Sakata I, Kusano S. Primary peritoneal clear cell carcinoma. J Comput Assist Tomogr2004 Sep-Oct;28(5):617-9.

14. Ichimura T, Ishiko O, Nishimura S, Kojima T, Shimura K. Primary peritoneal clear cell carcinoma: excellent results from paclitaxel and carboplatin combination chemotherapy. Oncol Rep2001 Nov-Dec;8(6):1243-5.

15. Lee KR, Verma U, Belinson J. Primary clear cell carcinoma of the peritoneum. Gynecol Oncol1991 Jun;41(3):259-62.

16. Muezzinoglu B, Corak S, Yucesoy I. Primary peritoneal clear cell adenocarcinoma associated with endometriosis. Appl Immunohistochem Mol Morphol2011 Jul;19(4):384-5.

17. Takano M, Yoshikawa T, Kato M, Aida S, Goto T, Furuya K, Kikuchi Y. Primary clear cell carcinoma of the peritoneum: report of two cases and a review of the literature. Eur J Gynaecol Oncol2009;30(5):575-8.

18. Terada T, Kawaguchi M. Primary clear cell adenocarcinoma of the peritoneum. Tohoku J Exp Med2005 Jul;206(3):271-5.

19. Tziortzioti V, Apessou D, Antoniou S, Giantzoglou A, Paissios P. Clear cell adenocarcinoma of the peritoneum associated with clear cell adenocarcinoma arising in an endometrial polyp. J Obstet Gynaecol1999 Sep;19(5):557-8.

20. Beriwal S, Demanes DJ, Erickson B, Jones E, De Los Santos JF, Cormack RA, Yashar C, Rownd JJ, Viswanathan AN. American Brachytherapy Society consensus guidelines for interstitial brachytherapy for vaginal cancer. Brachytherapy2012 Jan-Feb;11(1):68-75.

21. Dimopoulos JC, Petrow P, Tanderup K, Petric P, Berger D, Kirisits C, Pedersen EM, van Limbergen E, Haie-Meder C, Potter R. Recommendations from Gynaecological (GYN) GEC-ESTRO Working Group (IV): Basic principles and parameters for MR imaging within the frame of image based adaptive cervix cancer brachytherapy. Radiother Oncol2012 Apr;103(1):113-22.

22. Hu KS, Enker WE, Harrison LB. High-dose-rate intraoperative irradiation: current status and future directions. Semin Radiat Oncol2002 Jan;12(1):62-80.

23. Nag S, Hu KS. Intraoperative high-dose-rate brachytherapy. Surg Oncol Clin N Am2003 Oct;12(4):1079-97.

24. Chi DS, Zivanovic O, Levinson KL, Kolev V, Huh J, Dottino J, Gardner GJ, Leitao MM, Jr., Levine DA, Sonoda Y, Abu-Rustum NR, Brown CL, Barakat RR. The incidence of major complications after the performance of extensive upper abdominal surgical procedures during primary cytoreduction of advanced ovarian, tubal, and peritoneal carcinomas. Gynecol Oncol2010 Oct;119(1):38-42.

25. Langstraat C, Aletti GD, Cliby WA. Morbidity, mortality and overall survival in elderly women undergoing primary surgical debulking for ovarian cancer: a delicate balance requiring individualization. Gynecol Oncol2011 Nov;123(2):187-91.

26. Kim A, Schreiber D, Rineer J, Choi K, Rotman M. Impact of adjuvant external-beam radiation therapy in early-stage uterine papillary serous and clear cell carcinoma. Int J Radiat Oncol Biol Phys2011 Nov 15;81(4):e639-44.

27. Thomas M, Mariani A, Wright JD, Madarek EO, Powell MA, Mutch DG, Podratz KC, Dowdy SC. Surgical management and adjuvant therapy for patients with uterine clear cell carcinoma: a multi-institutional review. Gynecol Oncol2008 Feb;108(2):293-7.

28. Townamchai K, Lee LJ, Poorvu PD, Damato AL, Viswanathan AN. Vaginal brachytherapy for early stage uterine papillary serous and clear cell endometrial cancer. Gynecol Oncol2012 Dec 21.

29. Batchelor EC, Watkins JM, Jenrette JM, 3rd. Definitive radiotherapy for medically inoperable early-stage serous and clear cell uterine carcinoma. Radiat Med2007 Dec;25(10):536-40.

30. Varughese J, Hui P, Lu L, Yu H, Schwartz PE. Clear cell cancer of the uterine corpus: the association of clinicopathologic parameters and treatment on disease progression. J Oncol2011;2011:628084.

31. Batchelor EC, Watkins JM, Creasman WT, Kohler MF, Sinha D, Jenrette JM. The role of radiotherapy in the management of resected uterine papillary serous and clear cell carcinoma. Eur J Obstet Gynecol Reprod Biol2008 Dec;141(2):163-8.

32. Suzuki M, Saga Y, Tsukagoshi S, Tamura N, Sato I. Recurrent ovarian clear cell carcinoma: complete remission after radiation in combination with hyperthermia; a case study and in vitro study. Cancer Biother Radiopharm2000 Dec;15(6):625-8.

33. Takai N, Utsunomiya H, Kawano Y, Nasu K, Narahara H, Miyakawa I. Complete response to radiation therapy in a patient with chemotherapy-resistant ovarian clear cell adenocarcinoma. Arch Gynecol Obstet2002 Dec;267(2):98-100.

34. Goldberg MI, Belinson JL, Hutson ED, Nordqvist SR. Clear cell adenocarcinoma arising in endometriosis of the rectovaginal septum. Obstet Gynecol1978 Jan;51(1 Suppl):38s-40s.

35. Bats AS, Zafrani Y, Pautier P, Duvillard P, Morice P. Malignant transformation of abdominal wall endometriosis to clear cell carcinoma: case report and review of the literature. Fertil Steril2008 Oct;90(4):1197 e13-6.

36. Hitti IF, Glasberg SS, Lubicz S. Clear cell carcinoma arising in extraovarian endometriosis: report of three cases and review of the literature. Gynecol Oncol1990 Dec;39(3):314-20.

37. ICRU Report No. 38. Dose and volume specification for reporting intracavitary therapy in gynecology., (1985).

**Figure Legends**


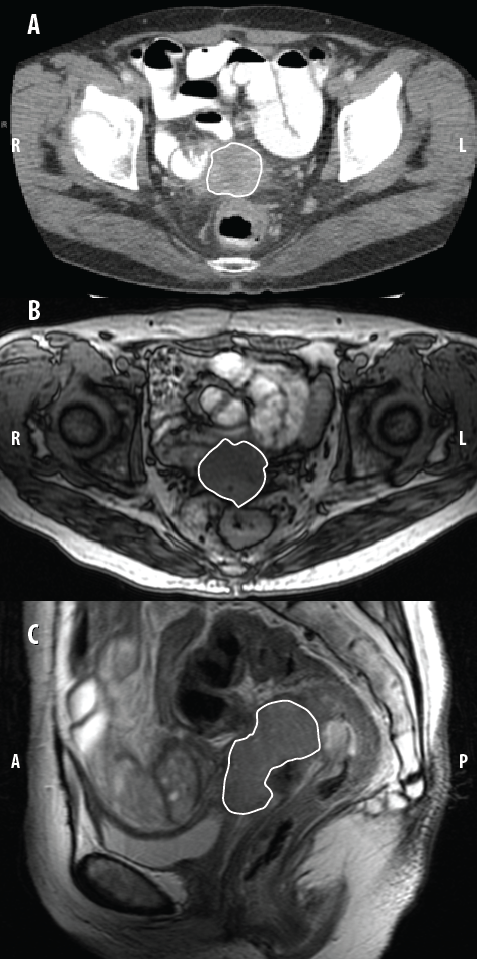


Fig. 1. Diagnostic imaging. (A) Axial CT image of the pelvis obtained at presentation shows a supravaginal 3.7 x 3.9 cm primary peritoneal clear cell carcinoma (PP-CCC) and an (B) axial and (C) sagittal T2-weighted MR image of the pelvis obtained one month following presentation which shows a supravaginal 5.6 x 3.7 x 3.5 cm primary peritoneal clear cell carcinoma. The lesion appears inseparable from the anterior wall of the proximal rectum.


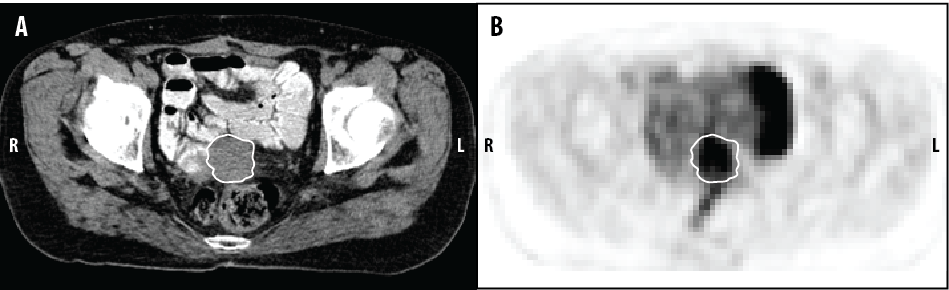


Fig. 2. Post-chemotherapy (A) axial CT and (B) PET image of the pelvis shows a residual 3.3 cm prerectal/primary peritoneal clear cell carcinoma with FDG activity in the posterior vagina.


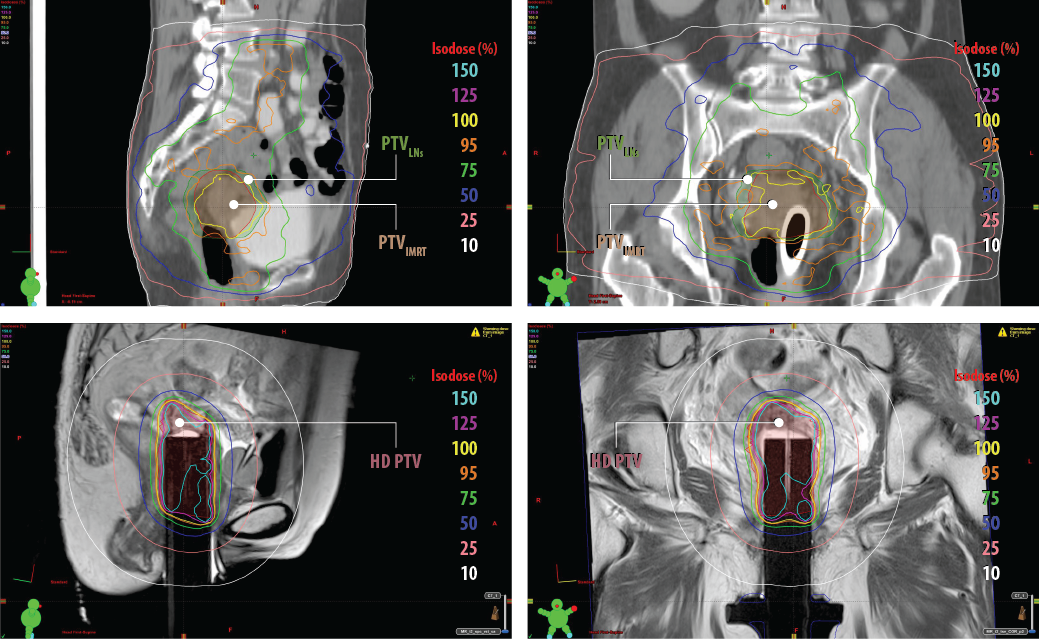


(b)

(d)

(c)

(a)

Fig. 3. Isodose distributions on the (a) parasagittal and (b) paracoronal CT view for the approved external beam radiotherapy and (c) parasagittal and (d) paracoronal T2-weighted MR view for the approved HDR brachytherapy treatment plan. The external beam PTV_IMRT_ and PTV_LNs_ volumes are shown in the shadowed red and green contours and the HDR PTV volume is shown in the shadowed red contour.


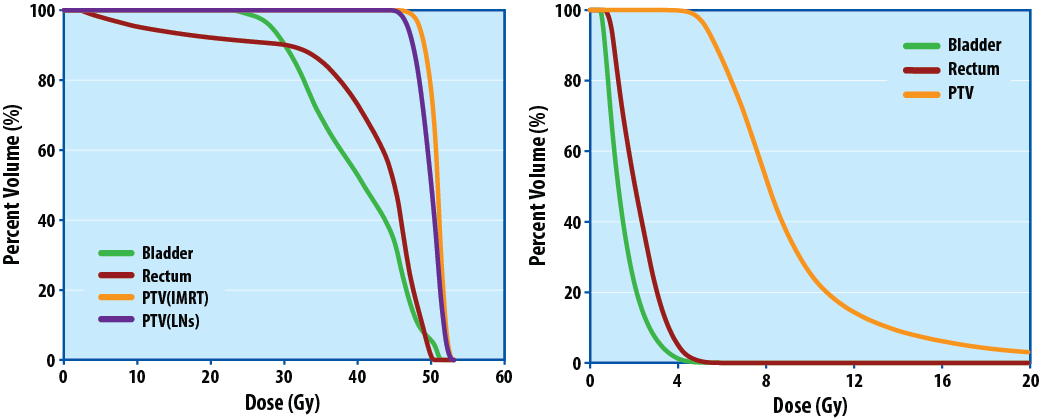


Fig. 4. Cumulative dose volume histograms for (a) external beam and (b) high-dose-rate brachytherapy plans.


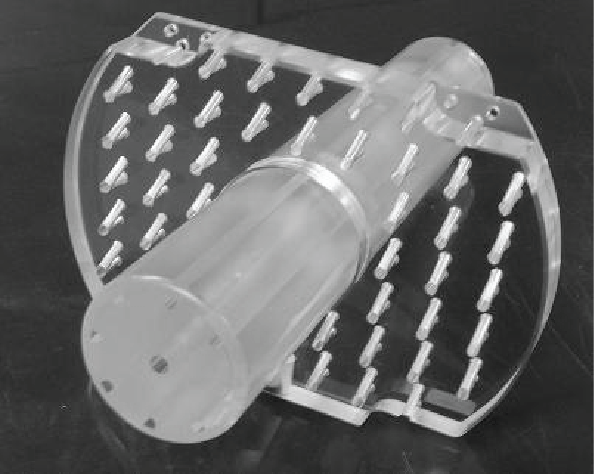


Fig. 5. A custom 30 millimeter diameter interstitial vaginal cylinder with perineal template.


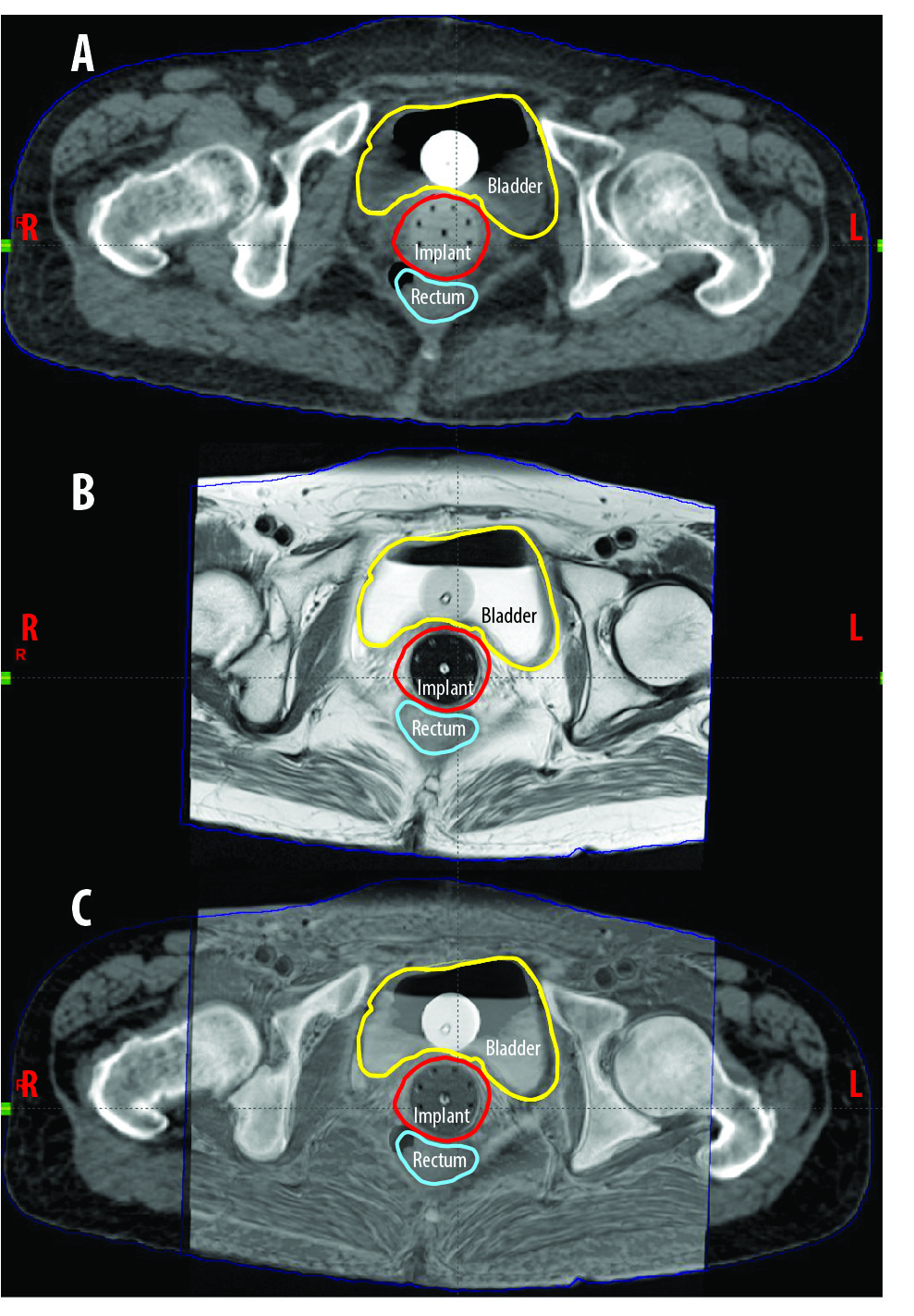


Fig. 6. (A) CT, (B) T2 weighted MR, and (C) registered CT/MR axial images of the custom interstitial brachytherapy cylinder at time of planning simulation. The yellow, red, and blue outlines represent the bladder, PTV, and rectum contours, respectively.


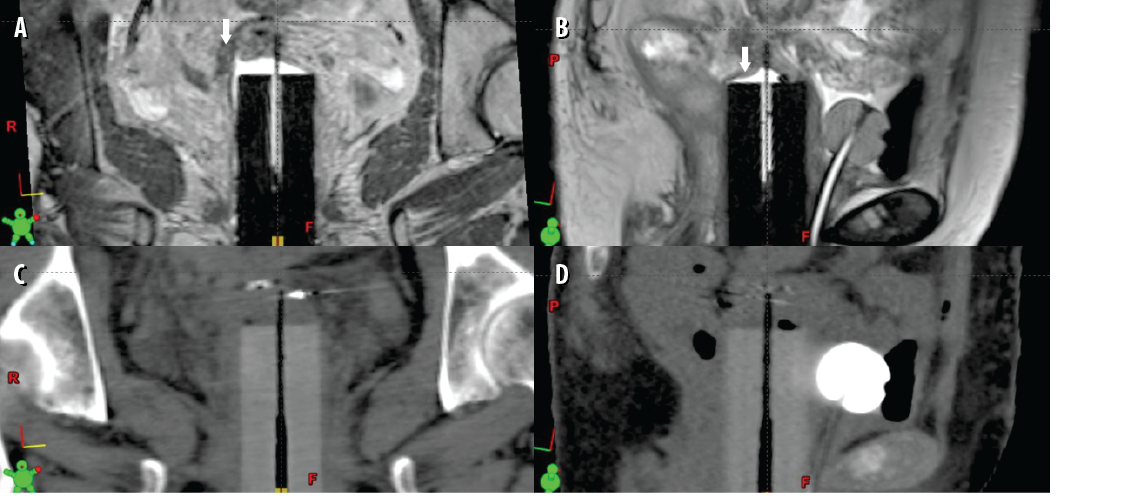


Fig. 7. (A) & (C) Paracoronal and (B) & (C) parasagittal T2 weighted MR and CT, respectively, through the custom interstitial applicator.


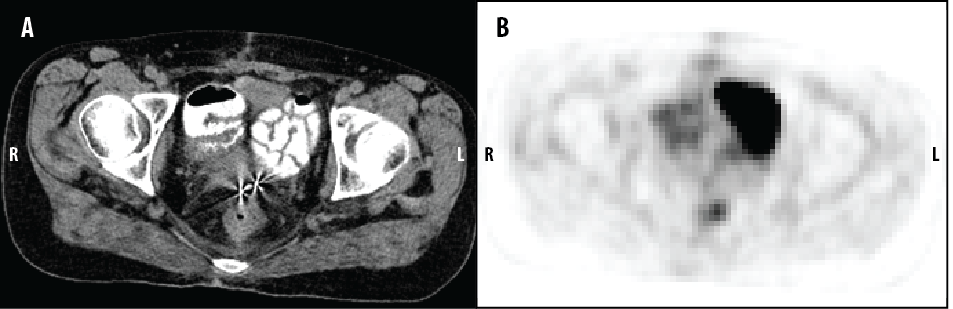


Fig. 8. Post radiotherapy (A) CT and (B) PET image of the pelvis obtained 4 months following radiotherapy shows near complete resolution of tumor.

Table 1. Summary of the dose quality parameters for the EBRT (PTV_IMRT_) and HDR treatment plans.

| Dose Quality Parameter | EBRT | HDR | EQD2 (Gy_2_)* | |
| --- | --- | --- | --- | --- |
|  |  |  | EBRT | HDR |
| PTV V100 (%) | 61.60 | 97.36 | ---- | ---- |
| PTV V95 (%) | 96.54 | 98.68 | ---- | ---- |
| PTV V90 (%) | 99.98 | 99.42 | ---- | ---- |
| PTV D100 (Gy) | 44.22 | 9.45 | 43.4 | 10.4 |
| PTV D95 (Gy) | 47.74 | 15.81 | 47.4 | 20.1 |
| PTV D90 (Gy) | 48.40 | 17.1 | 48.1 | 22.4 |
| Bladder D2cc (Gy) | 50.49 | 11.85 | 50.7 | 16.5 |
| Bladder point** (Gy) | N/A | 13.02 | ---- | 19.1 |
| Rectum D2cc (Gy) | 49.28 | 11.94 | 49.0 | 16.7 |

*Additionally, the equivalent 2 Gy dose, EQD2, has been calculated assuming an α/β of 10 for the PTV and 3 for the bladder and rectum.

**The bladder point was positioned based on the ICRU 38 ([37](#_ENREF_37)).
